# *PKD1* mutation may epistatically ameliorate nephronophthisis progression in patients with *NPHP1* deletion

**DOI:** 10.1002/ccr3.1947

**Published:** 2019-01-09

**Authors:** Saki Watanabe, Jun Ino, Takuya Fujimaru, Sekiko Taneda, Taro Akihisa, Shiho Makabe, Hiroshi Kataoka, Takayasu Mori, Eisei Sohara, Shinichi Uchida, Kosaku Nitta, Toshio Mochizuki

**Affiliations:** ^1^ Department of Medicine, Kidney Center Tokyo Women’s Medical University Shinjuku‐ku Japan; ^2^ Department of Medicine, Division of Nephrology Todachuo General Hospital Toda Japan; ^3^ Department of Nephrology, Graduate School of Medical and Dental Sciences Tokyo Medical and Dental University Bunkyo‐ku Japan; ^4^ Department of Pathology Tokyo Women’s Medical University Shinjuku‐ku Japan; ^5^ Clinical Research Division for Polycystic Kidney Disease, Department of Medicine, Kidney Center Tokyo Women’s Medical University Shinjuku‐ku Japan

**Keywords:** ADPKD, ciliopathy, epistatic effect, nephronophthisis, polycystic kidney disease

## Abstract

We report a patient with adult‐onset nephronophthisis (NPHP) that was identified a homozygous full gene deletion of *NPHP1 *and a heterozygous *PKD1 *mutation. We suggest that the *PKD1* mutation may have epistatically ameliorated NPHP disease progression and that the screening of larger cohorts for similar possible epistatic effects is needed.

## 1 INTRODUCTION

Nephronophthisis (NPHP) is a progressive renal disease that onsets during childhood or adolescence and presents with a range of clinical characteristics including polydipsia,polyuria, and secondary enuresis, that cause patients to progress to end‐stage renal disease (ESRD).^1^ To date, more than 20 genes have been implicated to cause NPHP, which is predominantly inherited in an autosomal recessive manner.^2^, ^3^ *NPHP* genes encode proteins expressed by the primary cilia of the renal tubular epithelium and mediate intercellular and cell‐extracellular matrix signaling, cell adhesion, and cell polarity. In fact, NPHP is considered to be a ciliopathy, since it is caused by structural and functional damage of primary cilia, basal body, and centrosome functions.^3^, ^4^

NPHP patients are classified as either infantile, juvenile, or adolescent‐type NPHP according to the age of onset. Specifically, infantile NPHP onsets between 0 and 3 years, whereas the average age of onset for juvenile and adolescent‐type NPHP is 13 and 19 years, respectively.^1^ While most patients with *NPHP1‐*associated juvenile NPHP progress to ESRD by an average age of 18 years, a recent study reported that some do not exhibit ESRD until they reach adulthood.^5^

Autosomal dominant polycystic kidney disease (ADPKD) has similarly been shown to be a ciliopathy that is characterized by the progressive development and accumulation of numerous renal cysts, eventually leading to ESRD.^6^ ADPKD is caused by mutations in *PKD1* and *PKD2, *which encode polycystin1 (PC1) and polycystin 2 (PC2) proteins, respectively, that are expressed in the cilia of renal tubular cells. PC1 normally functions to sense the flow of urine, and then transmits a signal to PC2, which is an intracellular calcium release channel. The resulting PC2‐mediated influx of calcium regulates the renal tubular diameter. Impairment of these functions by ADPKD‐causative mutations leads to cyst formation.

Herein, we report a patient with adult‐onset NPHP‐induced ESRD that was identified to harbor a homozygous full gene deletion of *NPHP1 *and a heterozygous *PKD1 *substitution.

## 2 CASE REPORT

The patient was a 34‐year‐old Japanese man, whose family history included a father with hypertension, and a mother that suffered a subarachnoid hemorrhage, but which did not include either a consanguineous marriage or any incidence of renal cysts. At the age of 31, the patient presented with mild albuminuria, and a serum creatinine (Cr) level of 2.98 mg/dL. At the age of 34, the patient was admitted to hospital with a creatinine level of 8.2 mg/dL.

**Figure 1:**
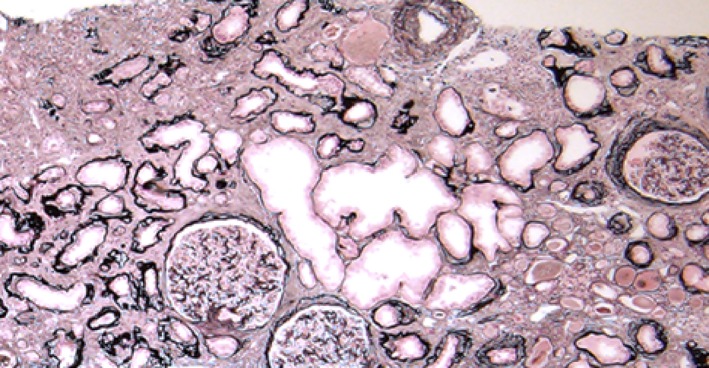
Evaluation of renal biopsy specimens using light microscopy. The patients’ (periodic acid‐Schiff‐stained) renal biopsy specimens displayed thickening of the renal tubular basement membrane (TBM), tubular atrophy, and tubulointerstitial fibrosis with chronic inflammation. As shown here, some tubules were found to be dilated, and the glomeruli were unaffected by the observed changes. Furthermore, the TBM can be seen to be irregularly thickened/thinned in certain areas, and there are numerous sections showing a transition between normal and thickened or thinner sections. TBM duplication was also evident. (Original magnification, ×40)

**Figure 2:**
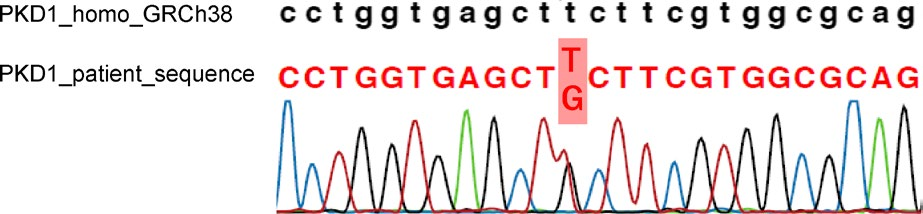
Sequencing chromatogram showing the patient's heterozygous substitution (c. 6395T>G, p.Phe2132Cys) in exon 15 of *PKD1*. Upper sequence, reference human *PKD1* sequence; lower sequence, patient mutant *PKD1* sequence

Upon admission, the patient's height, weight, and blood pressure were 179 cm, 58 kg, and 126/80 mm Hg, respectively. The patient exhibited anemia (Hb 8.2 g/dL), azotemia (Cr 8.38 mg/dL), hyperphosphatemia, metabolic acidosis, and secondary hyperparathyroidism; thus, he was diagnosed with ESRD. An abdominal ultrasonography revealed that, although the size of both kidneys appeared to be normal, the patient's renal parenchyma showed increased brightness. We performed a percutaneous renal biopsy; histologically, six of 16 glomeruli displayed global sclerosis, along with mild cellular infiltration, conspicuous interstitial fibrosis, renal tubular atrophy, and cystoid irregular dilation (Figure 1), suggesting an NPHP diagnosis. We performed targeted sequencing using a next‐generation sequencer,^7^ with the approval by the research ethics committee of Tokyo Medical and Dental University in accordance with the Declaration of Helsinki and the patient's written informed consent. A homozygous full gene deletion of *NPHP1* (NM_000272.3:g110879716‐110962709) was resultantly identified, as well as heterozygous substitutions in *PKD1* (NM_0001009944.2:c.6395T>G(p.Phe2132Cys)) (Figure 2), *BBS1 *(NM_024649.4:c.908T>C(p.Val303Ala))*,* and *INPP5E *(NM_019892.4:c.1652C>T(p.Thr551Met))*.*

## 3 DISCUSSION

The genetic analyses in our adult‐onset patient revealed a homozygous full gene deletion of*NPHP1*, which is typically associated with juvenile nephronophthisis.^3^, ^4^, ^8^, ^9^ Albeit rare, other similar patients of NPHP‐induced delayed/adult‐onset ESRD have previously been observed.^10^, ^11^, ^12^, ^13^ More recently, 26 (0.5%) patients harboring a homozygous *NPHP1* full gene deletion were identified among 5606 patients that underwent kidney transplantation.^5^ The average age at which these patients developed ESRD was 30 years (range, 18‐61 years), and 54% of the patients were aged greater than 30 years at ESRD onset. In many of these previous reports, the presence of NPHP‐modifier genes was discussed as a probable cause of the late ESRD onset. In addition to the identified *NPHP1 *full gene deletion, the patient was also shown to harbor heterozygous substitutions in *PKD1, BBS1,* and *INPP5E*. The *PKD1* substitution (p.Phe2132Cys) (dbSNP: rs150154235, https://www.ncbi.nlm.nih.gov/projects/SNP/snp_ref.cgi?rs=150154235, https://www.ncbi.nlm.nih.gov/clinvar/variation/431936/) identified in this patient was previously reported in the PKD Mutation Database as a “likely pathogenic mutation”.^14^ Although the detected *BBS1* substitution (p.Val303Ala) (rs750668484 in NCBI (https://www.ncbi.nlm.nih.gov/projects/SNP/snp_ref.cgi?rs=750668484) has been previously reported as a heterozygous mutation (AGVGD class, C25; SIFT prediction, deleterious; MAPP prediction, good), its pathological significance is not yet clear.^15^ Likewise, the identified *INPP5E* substitution (p.Thr551Met) is to date considered to be a variant of uncertain significance (dbSNP: rs75342839; https://www.ncbi.nlm.nih.gov/snp/75342839, https://preview.ncbi.nlm.nih.gov/clinvar/variation/373633/). Each of these genetic mutations may have modulated the clinical course of the patient's NPHP via incurred epistatic effects, as has been previously proposed for many other hereditary disorders and ciliopathies. Indeed, while autosomal recessive disorders develop as a result of homozygous or compound heterozygous mutations, their phenotypes are often modified by the presence of a third gene mutation. For example, a family patient of ciliopathic Bardet‐Biedl syndrome (BBS) was previously reported, in which affected members harbored causative mutations in *BBS1, BBS2, *and/or *BBS6*, as well as additional heterozygous mutations in the other *BBS *genes, which is considered to enhance the disease phenotype.^16^, ^17^ Further, studies using zebrafish have shown that heterozygous C430T mutations in *MGC1203*, which is a gene shown to be related to *BBS4*, similarly enhance the BBS phenotype.^18^ Likewise, in NPHP, additional heterozygous mutations in other *NPHP* genes have been shown to modulate the disease phenotype.^19^, ^20^, ^21^ Crossing homozygous *Nphp1*‐knockout (*Nphp* ^‐/‐^) with heterozygous *Ah1‐*knockout mice (*Ah1* ^+/−^) (the causative gene in Joubert syndrome) has been shown to enhance the development of retinal degenerative lesions.^22^

While the pathological significances of the *BBS1 *and *INPP5E* mutations are unknown, evidence suggests that the *PKD1 *mutation may be a contributing factor to NPHP disease progression. As an autosomal dominant disorder, ADPKD is induced by a heterozygous *PKD1 *mutation that is present in the whole body; however, a heterozygous *PKD1 *mutation does not lead to cyst formation because of the existence of normal allele of *PKD1*. When somatic mutations in the normal *PKD1* allele, that is proposed as “two‐hit theory”, occur in the renal tubular cells, PC1 function in the cells is lost, thereby leading to cyst formation.^6^

Although both nephrocystin 1, which is encoded by*NPHP1*, and PC1 function as ciliary proteins, they are also known to have cilia‐independent functions. Interestingly, a previous study showed that the polyproline motif‐2 present in the PC1 C‐terminal binds to the nephrocystin 1 SH3 domain to control the apoptosis of renal tubular epithelial cells.^23^ PC1 has also been shown to be capable of independently suppressing cell proliferation, without interacting with nephrocystin 1. We hypothesize that the occurrence of this independent function in the renal tubular cells may have acted as an epistatic protective effect in the present patient. Renal tubular cells harboring both the homozygous *NPHP1* deletion and heterozygous *PKD1* mutation would be unable to maintain controlled apoptosis; however, in the absence of any further somatic mutations, the remaining functional *PKD1* allele would be sufficient to suppress excess cell proliferation. The introduction of additional somatic *PKD1* mutations in even a small portion of the patient's renal tubular cells would induce a loss of PC1 function, rendering the cells unable to suppress proliferation, and thus, potentially leading to cell immortalization.^24^ Cellular functions not related to nephrocystin 1 and PC1 would be retained in these proliferated renal tubular cells, contributing to the maintenance of renal function, and thereby delaying the onset of ESRD.

In conclusion, we here describe a patient with delayed‐onset NPHP that was caused by a homozygous *NPHP1* full gene deletion, and possibly modified by heterozygous mutations in cyst formation‐related genes. An epistatic action induced via the detected *PKD1* mutation likely ameliorated NPHP disease progression. Although epistatic effects have regularly been reported in ciliopathies, we have no evidence of the mechanism of an ameliorating effect of the *PKD1* variant on the *NPHP1*‐null phenotype. Therefore, we need to validate this finding on larger cohorts of late onset NPHP type 1.

## CONFLICT OF INTEREST

None declared.

## AUTHOR CONTRIBUTIONS

SW: wrote the main part of the manuscript according to collected data and followed up with the patient. JI: helped to data interpretation and manuscript evaluation, also helped to follow up with the patient. TF, TM(Mori), ES and SU: performed genetic analysis and wrote “Genetic Assessments” part of the manuscript. ST: performed histopathological evaluations and wrote “Pathological Assessments” part of the manuscript. TA, SM, HK and KN: helped to evaluate and edit the manuscript. TM(Mochizuki): supervised development of work, helped in data interpretation and manuscript evaluation, revised the manuscript, and finally approved the version to be published.

## References

[1] Wolf MT , Hildebrandt F . Nephronophthisis. Pediatr Nephrol. 2011;26(2):181‐194. 20652329 10.1007/s00467-010-1585-z PMC4160028

[2] Srivastava S , Molinari E , Raman S , Sayer JA . Many Genes‐One Disease? Genetics of Nephronophthisis (NPHP) and NPHP‐Associated Disorders. Front Pediatr. 2017;5:287. 29379777 10.3389/fped.2017.00287 PMC5770800

[3] Hildebrandt F , Attanasio M , Otto E . Nephronophthisis: disease mechanisms of a ciliopathy. J Am Soc Nephrol. 2009;20(1):23‐35. 19118152 10.1681/ASN.2008050456 PMC2807379

[4] Braun DA , Hildebrandt F . Ciliopathies. Cold Spring Harb Perspect Biol. 2017;9:3. 10.1101/cshperspect.a028191 PMC5334254 27793968

[5] Snoek R , van Setten J , Keating BJ , et al. NPHP1 (Nephrocystin‐1) gene deletions cause adult‐onset ESRD. J Am Soc Nephrol. 2018;29(6):1772‐1779. 29654215 10.1681/ASN.2017111200 PMC6054334

[6] Mochizuki T , Tsuchiya K , Nitta K . Autosomal dominant polycystic kidney disease: recent advances in pathogenesis and potential therapies. Clin Exp Nephrol. 2013;17(3):317‐326. 23192769 10.1007/s10157-012-0741-0

[7] Fujimaru T , Mori T , Sekine A , et al. Kidney enlargement and multiple liver cyst formation implicate mutations in PKD1/2 in adult sporadic polycystic kidney disease. Clin Genet. 2018;94(1):125‐131. 29520754 10.1111/cge.13249

[8] Halbritter J , Porath JD , Diaz KA , et al. Identification of 99 novel mutations in a worldwide cohort of 1,056 patients with a nephronophthisis‐related ciliopathy. Hum Genet. 2013;132(8):865‐884. 23559409 10.1007/s00439-013-1297-0 PMC4643834

[9] Saunier S , Calado J , Benessy F , et al. Characterization of the NPHP1 locus: mutational mechanism involved in deletions in familial juvenile nephronophthisis. Am J Hum Genet. 2000;66(3):778‐789. 10712196 10.1086/302819 PMC1288163

[10] Hildebrandt F , Strahm B , Nothwang HG , et al. Molecular genetic identification of families with juvenile nephronophthisis type 1: rate of progression to renal failure. APN Study Group. Arbeitsgemeinschaft fur Padiatrische Nephrologie. Kidney Int. 1997;51(1):261‐269. 8995741 10.1038/ki.1997.31

[11] Bollee G , Fakhouri F , Karras A , et al. Nephronophthisis related to homozygous NPHP1 gene deletion as a cause of chronic renal failure in adults. Nephrol Dial Transplant. 2006;21(9):2660‐2663. 16782989 10.1093/ndt/gfl348

[12] Hoefele J , Nayir A , Chaki M , et al. Pseudodominant inheritance of nephronophthisis caused by a homozygous NPHP1 deletion. Pediatr Nephrol. 2011;26(6):967‐971. 21258817 10.1007/s00467-011-1761-9 PMC3342573

[13] Haghighi A , Savaj S , Haghighi‐Kakhki H , Benoit V , Grisart B , Dahan K . Identification of an NPHP1 deletion causing adult form of nephronophthisis. Ir J Med Sci. 2016;185(3):589‐595. 26037636 10.1007/s11845-015-1312-7

[14] O'Brien K , Font‐Montgomery E , Lukose L , et al. Congenital hepatic fibrosis and portal hypertension in autosomal dominant polycystic kidney disease. J Pediatr Gastroenterol Nutr. 2012;54(1):83‐89. 21694639 10.1097/MPG.0b013e318228330c PMC8366680

[15] Watson CM , El‐Asrag M , Parry DA , et al. Mutation screening of retinal dystrophy patients by targeted capture from tagged pooled DNAs and next generation sequencing. PLoS ONE. 2014;9(8):e104281. 25133751 10.1371/journal.pone.0104281 PMC4136783

[16] Katsanis N , Ansley SJ , Badano JL , et al. Triallelic inheritance in Bardet‐Biedl syndrome, a Mendelian recessive disorder. Science. 2001;293(5538):2256‐2259. 11567139 10.1126/science.1063525

[17] Badano JL , Kim JC , Hoskins BE , et al. Heterozygous mutations in BBS1, BBS2 and BBS6 have a potential epistatic effect on Bardet‐Biedl patients with two mutations at a second BBS locus. Hum Mol Genet. 2003;12(14):1651‐1659. 12837689 10.1093/hmg/ddg188

[18] Badano JL , Leitch CC , Ansley SJ , et al. Dissection of epistasis in oligogenic Bardet‐Biedl syndrome. Nature. 2006;439(7074):326‐330. 16327777 10.1038/nature04370

[19] Tory K , Lacoste T , Burglen L , et al. High NPHP1 and NPHP6 mutation rate in patients with Joubert syndrome and nephronophthisis: potential epistatic effect of NPHP6 and AHI1 mutations in patients with NPHP1 mutations. J Am Soc Nephrol. 2007;18(5):1566‐1575. 17409309 10.1681/ASN.2006101164

[20] Hoefele J , Wolf MT , O'Toole JF , et al. Evidence of oligogenic inheritance in nephronophthisis. J Am Soc Nephrol. 2007;18(10):2789‐2795. 17855640 10.1681/ASN.2007020243

[21] Penchev V , Boueva A , Kamenarova K , et al. A familial case of severe infantile nephronophthisis explained by oligogenic inheritance. Eur J Med Genet. 2017;60(6):321‐325. 28392475 10.1016/j.ejmg.2017.04.002

[22] Louie CM , Caridi G , Lopes VS , et al. AHI1 is required for photoreceptor outer segment development and is a modifier for retinal degeneration in nephronophthisis. Nat Genet. 2010;42(2):175‐180. 20081859 10.1038/ng.519 PMC2884967

[23] Wodarczyk C , Distefano G , Rowe I , et al. Nephrocystin‐1 forms a complex with polycystin‐1 via a polyproline motif/SH3 domain interaction and regulates the apoptotic response in mammals. PLoS ONE. 2010;5(9):e12719. 20856870 10.1371/journal.pone.0012719 PMC2939065

[24] Nishio S , Hatano M , Nagata M , et al. Pkd1 regulates immortalized proliferation of renal tubular epithelial cells through p53 induction and JNK activation. J Clin Investig. 2005;115(4):910‐918. 15761494 10.1172/JCI22850 PMC1059447

